# Evaluation of the impact of shigellosis exclusion policies in childcare settings upon detection of a shigellosis outbreak

**DOI:** 10.1186/s12879-019-3796-7

**Published:** 2019-02-19

**Authors:** Cristina Carias, Eduardo A. Undurraga, Jacqueline Hurd, Emily B. Kahn, Martin I. Meltzer, Anna Bowen

**Affiliations:** 1 National Center for Emerging and Zoonotic Infectious Diseases, Centers for Disease Control and Prevention, 1600 Clifton Road, H24-11, Atlanta, GA 30329-4027 USA; 2 Escuela de Gobierno, Pontificia Universidad Católica de Chile, Santiago, Región Metropolitana Chile

**Keywords:** Shigellosis, Exclusion policies, Childcare settings

## Abstract

**Background:**

In the event of a shigellosis outbreak in a childcare setting, exclusion policies are typically applied to afflicted children to limit shigellosis transmission. However, there is scarce evidence of their impact.

**Methods:**

We evaluated five exclusion policies: Children return to childcare after: i) two consecutive laboratory tests (either PCR or culture) do not detect *Shigella*, ii) a single negative laboratory test (PCR or culture) does not detect *Shigella*, iii) seven days after beginning antimicrobial treatment, iv) after being symptom-free for 24 h, or v) 14 days after symptom onset. We also included four treatments to assess the policy options: i) immediate, effective treatment; ii) effective treatment after laboratory diagnosis; iii) no treatment; iv) ineffective treatment. Relying on published data, we calculated the likelihood that a child reentering childcare would be infectious, and the number of childcare-days lost per policy.

**Results:**

Requiring two consecutive negative PCR tests yielded a probability of onward transmission of < 1%, with up to 17 childcare-days lost for children receiving effective treatment, and 53 days lost for those receiving ineffective treatment.

**Conclusions:**

Of the policies analyzed, requiring negative PCR testing before returning to childcare was the most effective to reduce the risk of shigellosis transmission, with one PCR test being the most effective for the least childcare-days lost.

**Electronic supplementary material:**

The online version of this article (10.1186/s12879-019-3796-7) contains supplementary material, which is available to authorized users.

## Background

Shigellosis is an infectious disease characterized by diarrhea, stomach cramps, and sometimes fever, starting 1–3 days after exposure to *Shigella* bacteria (shigellae), typically lasting 5–7 days if untreated. Antimicrobial medications can be used to reduce the duration of severe cases [1]. Shigellosis is diagnosed by laboratory testing of the stools of an infected person. Shigellae are transmitted via the fecal-oral route, and ingestion of as few as 10 bacteria can cause infection. Every year ~ 500,000 cases of shigellosis occur in the United States; outbreaks are common in childcare settings and schools [1, 2].

To try to limit transmission, state policies commonly prevent children with shigellosis from attending childcare for specified periods of time after symptoms resolve and/or following one or more negative laboratory tests. These policies affect childcare attendance and income (e.g., productivity losses of caregivers), and present a burden to schools, healthcare providers, and local public health departments. However, there is limited evidence on the impact of exclusion policies on shigellosis transmission.

We evaluated the impact of five different child exclusion policies on the likelihood that a child returning to childcare would still be infectious, and on childcare-days lost for the afflicted children, upon the detection of a shigellosis outbreak. For each exclusion policy, we quantified the probability that children with shigellosis remained infectious upon school readmission (thus posing a risk of onward transmission) and the number of childcare-days lost. Our analysis provides evidence to inform policy decisions. We also provide a user-friendly spreadsheet tool with adjustable parameters as supplementary material for public use.

## Methods

We defined childcare as a facility that provides care and educational activities for around 45 children aged approximately 5 years or younger for several hours per day but not 24 h per day. We evaluated five childcare exclusion policies, reflecting policies currently used in various states (see Additional file 1: Appendix A) and policies from expert opinion (Table 1, Panel I). Because options for shigellosis management can have different levels of effectiveness, we evaluated each policy using four illustrative treatment scenarios for children with shigellosis (Table 1, Panel II). For each policy-treatment pair, we calculated the likelihood of an infectious child reentering childcare, and the expected number of childcare-days lost per child. To deal with uncertainty we included lower and upper bounds for all parameter values. Data were drawn from the literature when available, and from expert opinion when not (Table 2); the final results were calculated in 2017. Additional file 1: Appendix A and Additional file 2: Appendix B show the calculations for the probability of being infectious upon readmission to childcare.

**Table 1:** Main scenarios evaluated: shigellosis exclusion policies assessed and patient treatment scenarios

Main scenarios for evaluation	Definition
I. Shigellosis exclusion policies^a^	The patient can return to childcare if in compliance with the following requirements:
Two consecutive tests: Culture	Two consecutive laboratory culture analyses of convalescent^b^ stool samples yield negative results for *Shigella*
Two consecutive tests: PCR^c^	Two consecutive laboratory PCR analyses of convalescent^b^ stool samples yield negative results for *Shigella*
One test: Culture	One laboratory culture analysis of convalescent^b^ stool samples yields negative results for *Shigella*
One test: PCR	One laboratory PCR analysis of convalescent^b^ stool samples yields no *Shigella*
14 days after onset	14 days after symptom onset, with no tests performed during convalescence
7 days after beginning treatment	Seven days after beginning antimicrobial treatment, with no tests performed during convalescence
24 h symptom-free	24 h symptom-free, with no tests performed during convalescence
II. Treatment scenarios for each exclusion policy^d^	
A. Immediate, effective treatment	Child visits healthcare provider and starts effective antimicrobial treatment without requiring any further diagnosis or test on the second day of illness
B. Effective treatment after diagnosis	Child visits healthcare provider on the second day of illness, gets a stool culture with antimicrobial susceptibility testing, and starts effective treatment after results are available^e^
C. Ineffective treatment	Child visits healthcare provider and starts ineffective antimicrobial treatment on second to fourth day of illness
D. No treatment	Child receives no antimicrobial treatment^f^

Notes

^a^In all scenarios, the patient would get an initial diagnostic test to confirm *Shigella* infection

^b^Definition of convalescent stool samples: samples collected at least 24 h after completing antimicrobial therapy and/or diarrhea resolution; if performing more than 1 test, the samples should be collected at an interval of ≥24 h

^c^PCR: polymerase chain reaction

^d^The exclusion policy “7 days after beginning treatment” was not evaluated for the treatment scenario “D. No Treatment” given that they are mutually exclusive

^e^We assumed that the interval between starting treatment between patients receiving Treatment A and patients receiving Treatment B was two days. This includes the time necessary to do an additional test, receive the results, and have the doctor do a prescription for treatment after the first medical encounter

^f^For example, if the patient does not follow up after the initial diagnostic visit, or no antimicrobials are prescribed

**Table 2:** Parameters used to estimate the effects of different exclusion policies

Parameter	Value	Range	Source
Shedding duration (days)			
Treatment scenario A^a^	3.6	1–5	[3, 4]
Treatment scenario B^b^	5.6	3–7	[3, 4]
Treatment scenario C	31	17–41	[5, 6]
Treatment scenario D	11	4–31	[5, 6, 8]
Symptom duration			
Treatment scenario A	3.6	1–5	[3, 4]
Treatment scenario B	5.6	3–7	[3, 4]c
Treatment scenario C	10^d^	1–41	[3, 7]
Treatment scenario D^e^	5	1–30	[3, 4]
Days between doing test and receiving results from convalescent test (PCR)^f^	2	1–6	Expert opinion
Days between doing test and receiving results from convalescent test (Culture)^f^	3	2–7	Expert opinion
Duration of antimicrobial treatment (days)	5		[9]
Attack rate (%)^g^	25		[10, 11, 16, 17]
Test sensitivity PCR (%)^h^	96	94–98	[18]
Test sensitivity, stool culture (%)	52	44–72	[19, 20]
Test specificity, PCR or stool culture (%)^i^	95	90–99	[18, 20]

Notes

Different treatments: A. Immediate, effective treatment; B. Effective treatment after diagnosis; C. Ineffective treatment; D. No treatment

^a^We assumed that duration of fecal shedding of shigellae for patients treated with an appropriate antibiotic is similar to the duration of shigellosis symptoms

^b^Same assumption for the duration of fecal shedding of shigellae as for scenario A plus two days, which correspond to the assumed interval between starting treatment immediately after seeking care (Treatment A), and starting treatment after doing the test and receiving diagnosis (Treatment B)

^c^The symptom duration was estimated as scenario A plus two days

^d^The mean value of symptom duration for scenario C was assumed

^e^The upper bound of symptom duration for scenario D was based on data from State Health Departments and PulseNet outbreak 1407MLJ16–2

^f^Assumption based on the information provided by State Health Departments. For PCR tests, the lower bound for the time elapsed between receiving results from the first and second test is one day if the health department initiates the request. The upper bound was defined as six days if there are delays or a weekend between laboratory tests. One day was added for culture tests

^g^Hoffman et al. [10] estimated an overall attack rate of 25% in a Denver child-care servicing 18 months to 6 years old. The other references are used for suggested ranges in Additional file 2: Appendix B

^h^Proportion of tests from patients with shigellosis that show positive results (true positive rate)

^i^Proportion of tests from patients without shigellosis that show negative results (true negative rate). We assumed that the lower bound and median value of the test specificity were 90 and 95%

We estimated the likelihood that shigellosis patients returned to childcare while still infectious as well as the number of childcare-days lost per child (where all days lost are assumed to be childcare-days lost, not accounting for holidays or weekends), for policies based on convalescent stool tests (policies that excluded children until they had one or two consecutive negative stool tests with PCR or culture-based tests on specimens that were collected at least 24 h after completing antimicrobial therapy and/or diarrhea resolution). We evaluated test-based policies taking into account the sensitivity of PCR- and culture-based tests. Similarly, we evaluated policies that excluded children for fixed periods of time; these included exclusions for 14 days after symptom onset, 7 days after starting treatment, and 24 h after becoming symptom-free [3–8].

We evaluated each policy for patients undergoing different treatment scenarios, namely: A) immediate, effective treatment; B) effective treatment after diagnosis; C) ineffective treatment; D) no treatment. We considered as effective treatment the receipt of a course of antibiotics that the particular strain of *Shigella* bacteria was susceptible to, as recommended in the latest guidelines [9]. Ineffective treatment was defined as receiving an antibiotic that the *Shigella* strain was not susceptible to, or that did not have an effect in vivo based on pharmacokinetics [9].

For policies based on convalescent stool tests, the estimated likelihood that the returning patient reentered school while still infectious was calculated using the probability that the test would provide a false negative result, which was related to the test’s sensitivity (Additional file 1: Appendix A). We opted for this conservative estimate given variability in shedding shigellae in stool, symptom duration, and time interval before receiving the test. For policies based on a fixed time interval, the estimated likelihood that the patient returned to school while still infectious was based on the duration of shedding; in particular, we assumed that the proportion of children who remained infectious decreased linearly with each day of the shedding period, from 100% in the first day to 0% in the last day. The duration of the shedding period was estimated from the literature (Table 2).

For policies relying on negative stool sample results, the number of childcare-days lost was estimated by the number of days required to receive testing results, taking into account treatment duration, and shedding duration for policies based on convalescent stool tests. In particular, for these policies the number of childcare-days lost was calculated as a weighted average of the number of days required to obtain the required (one or two consecutive) negative results for *Shigella* for infectious children, and the number of days required to obtain said results for non-infectious children. The weights consisted of the likelihood of the child being infectious and non-infectious upon return to childcare. For policies based on fixed time intervals, we estimated the number of childcare-days lost by using said intervals.

We further explored the impact of exclusion policies in aggregated childcare-days lost (childcare-days lost for a group of children, assuming different children receive different treatments) for a given childcare in a separate sensitivity analysis (Additional file 3: Appendix C). We estimated the number of aggregated childcare-days lost as the attack rate multiplied by the setting size and by the number of childcare-days lost for a given combination of treatments the children receive. We considered a population of 45 children (equivalent to a small childcare facility), an attack rate of 25% [10, 11] for our reference analysis, and three different combinations of treatments. We show the results for various combinations of treatment types for our reference population of children (Additional file 3: Appendix C).

## Results

**Fig. 1:**
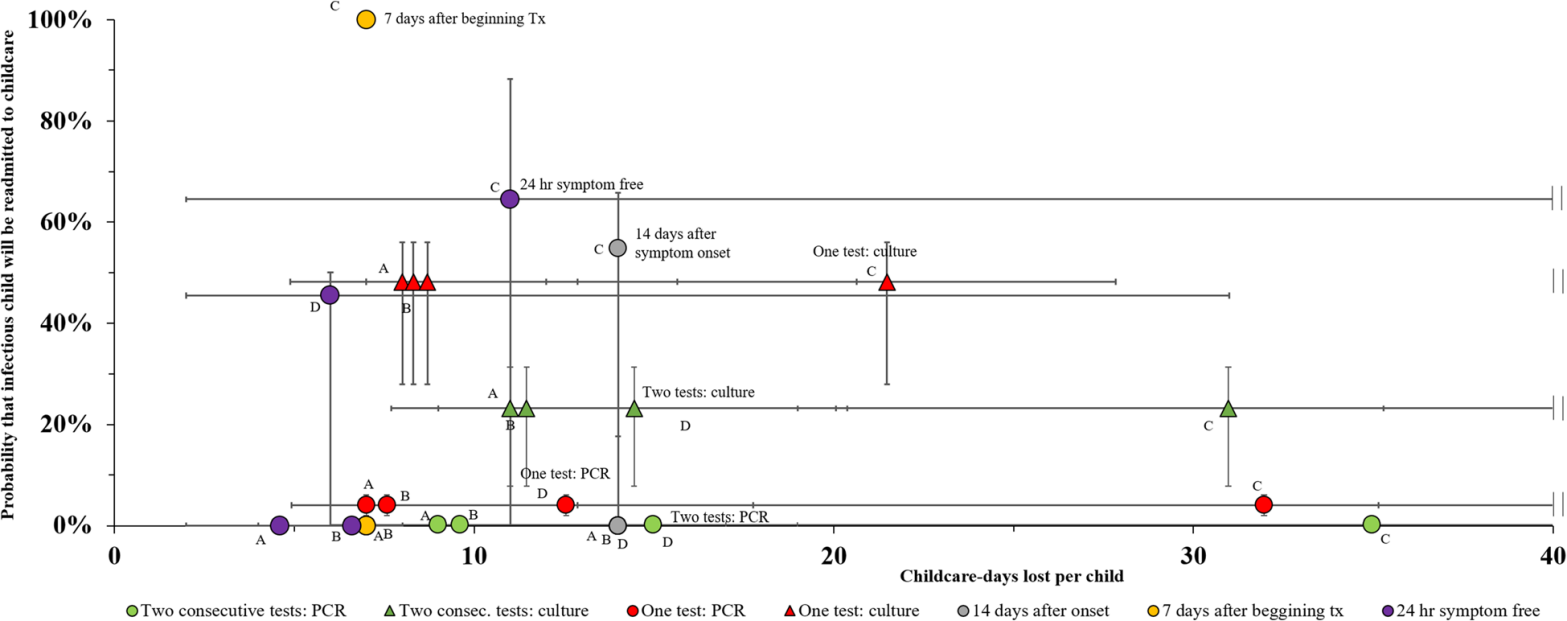
The impact of 7 different exclusion policies on childcare-days lost per child and probability of infectiousness upon readmission to childcare

Figure 1 shows the probability that an infectious child returns to childcare and the number of days that the child would be excluded, by treatment type and exclusion policy. The effectiveness of policies based on negative convalescent stool tests hinged on the test’s sensitivity, with PCR-based tests leading to lower probability of the child being infectious when readmitted. If the policy required 2 consecutive PCR-negative stool samples, the probability that the child returned to school infectious was < 1%, with the number of days the child spent at home ranging from 7 to 17 days (midpoint: 9 days) if the child received immediate, effective treatment. The maximum number of childcare-days lost per child increased to 19 days if the child received effective treatment after diagnosis; it was between 19 and 53 days if the child received ineffective treatment; and between 6 and 43 days if the child received no treatment. If only 1 *Shigella*-negative PCR test stool sample was required, the likelihood the child returned to school infectious was ≤6% and the number of days the child spent at home varied between 6 and 11 days (midpoint: 7 days) if the child received immediate, effective treatment; it was up to 13 days if the child received effective treatment after diagnosis; between 18 and 45 days for children receiving ineffective treatment; and between 5 and 35 days for children receiving no treatment.

While the specificity of PCR and stool culture tests is the same, PCR tests are almost twice as sensitive as stool cultures (Table 2). Thus, the type of diagnostic had a larger impact on readmission of infectious children than the number of tests performed. We estimated that the likelihood of reentering school while infectious after one negative PCR test was 2 to 6%, compared with 8 to 31% for two consecutive negative stool cultures. If only one negative culture was required, the likelihood that the child returned to school infectious ranged from 28 to 56%. The number of childcare-days lost per child for the exclusion policy involving one negative culture varied from 7 to 12 days for children receiving immediate, effective treatment, and from 16 to 28 days if the child received ineffective treatment. If two negative cultures were required, this interval ranged from 9 to 19 days if the child received immediate, effective treatment and from 20 to 44 days if the child received ineffective treatment.

The policy permitting readmission 7 days after beginning antimicrobial treatment showed minimal childcare-days lost for minimum risk (0%) of infectious child readmission if the antimicrobial treatment was effective. However, all (100%) children would be readmitted while infectious if they received inappropriate treatment, because the shedding duration would be longer than 7 days after beginning antimicrobial treatment. The risk of readmitting infectious students was very variable for policies in which children returned to school 14 days after symptom onset or 24 h after being symptom-free for patients receiving ineffective (Range: 0–88%) or no treatment (Range:0–50%).

In Additional file 3: Appendix C, we further explored variation in the aggregated childcare-days lost in a shigellosis outbreak for each exclusion policy, considering a setting of 45 children and an assumed treatment mix of affected children. Results show that the cost comparison (in aggregated childcare-days lost) hinged on treatment effectiveness. When the percent of patients receiving effective treatment increases, the estimated number of aggregated childcare-days lost decreases. Conversely, when the share of patients receiving ineffective or no treatment increases, the estimated number of aggregated childcare-days lost increases.

## Discussion

Exclusion policies for shigellosis patients based on convalescent testing most consistently minimized the probability of readmitting an infectious child to childcare, but varied in the number of childcare-days lost per child. PCR tests minimized the likelihood of an infectious child returning to childcare and the number of days the child was excluded. Given the different sensitivities of PCR and culture tests, the use of one PCR test more effectively minimized the probability of readmitting an infectious child than did two stool cultures. Policies based on a fixed number of exclusion days after an event (i.e., symptom onset, start of antimicrobial treatment, resolution of symptoms) exhibited greater variation for patients exposed to different treatments. Such policies resulted in fewer childcare-days lost per child only if a small percent of patients received ineffective treatment (e.g., treatment with an antimicrobial medication to which the *Shigella* strain was resistant).

Treatment using appropriate antimicrobial medications generally decreased the probability of readmitting an infectious child and the number of childcare-days per child lost compared with no treatment or ineffective antimicrobial treatment, and use of ineffective antimicrobials consistently maximized the probability of readmitting an infectious child and/or childcare-days lost per child. Overuse of antimicrobial treatment may also induce antimicrobial resistance, unnecessarily disrupt children’s microflora, and incur costs to the healthcare system and families. The prevalence of antimicrobial resistance is increasing among shigellae [12]; outbreaks of antimicrobial-resistant shigellosis may result in a high proportion of infectious children returning to childcare, extended days of exclusion, or both [13].

We evaluated child exclusion policies’ impact on the likelihood of readmitting infectious children to childcare and the number of childcare-days lost per child, assuming that the outbreak’s attack rate was unrelated to exclusion policies. In reality, exclusion policies that result in a higher likelihood of children returning to childcare while infectious may contribute to a higher attack rate, and thus higher number of childcare-days lost. That is, for policies resulting in a higher likelihood of returning to childcare while infectious, the current model may underestimate the number of childcare-days lost per child. While a dynamic infectious disease model would be required to quantify this bias, the current model serves as a lower bound of childcare-days lost (equivalent to assuming that after the initial outbreak is detected, caregivers’ alertness to the disease limits disease transmission, for instance, by being especially aware of symptoms in previously known patients).

Our results are also limited by the lack of data regarding the prevalence of different types of treatment and uncertainty about testing parameters. However, our supplementary material allows users to evaluate policies using new data or different assumptions. Another limitation is the absence of information regarding the timing of diagnosis and prevalence of different treatment methods. In our treatment scenarios, we opted not to mention the timing of diagnosis explicitly since the determining constraints for duration of infectiousness are the start and effectiveness of treatment, and health care providers may treat empirically in the absence of a laboratory diagnosis. To note, while culture-based diagnosis is slower to obtain than PCR-based diagnosis, culture-based diagnosis allows assessment of the resistance profile of the bacteria. Therefore, the type of diagnosis could be related to the likelihood of receiving ineffective treatment, which we have not considered. If this is the case, we may overestimate the advantages of testing via PCR.

Based on expert opinion, we assumed that children sought medical care on the second day of illness. Since the interval between symptom onset and care-seeking was assumed to be the same for children undergoing Treatments A-C, a delay in seeking medical care would shift the date of return to school equally forward for children undergoing treatment A-C relative to D. This would not affect the relative differences among most exclusion policies as applied to children undergoing most treatments. The only change in our estimates would be a relative increase in childcare-days lost per child to Treatment D, for policies requiring 24 h symptom-free or waiting 14 days after symptom onset. On another note, for the exclusion policy involving two consecutive tests, we assumed that the second test would be conducted upon receipt of the results of the first test. If the second test was conducted before the results of the first test were available, the number of childcare-days lost per child could be marginally less than we estimated.

Notably, these findings reflect scenarios with known shigellosis, such as during a shigellosis outbreak with laboratory-confirmed and epidemiologically linked cases. We assumed that the time-to-negative is equal among PCR and culture tests, which may not be the case. PCR tests may detect *Shigella* DNA after the bacteria are no longer viable, which would prolong childcare exclusion time, adding indirect costs to what is already a more expensive laboratory test (PCR tests cost about $27–$47; cultures cost about $9–$12 in 2015 USD [14, 15]).

## Conclusions

Of the policies analyzed, exclusion policies that most effectively reduced risk of shigellosis transmission in childcare settings included the use of PCR-based tests. Our estimation suggested that the type of test (PCR or culture) was more relevant than the number of tests performed, with one PCR test being more effective than 2 cultures. The performance of policies based on fixed time intervals (i.e., waiting 14 days after onset, 7 days after beginning treatment, or 24 h without symptoms) was a function of the effectiveness and timing of treatments. Given substantial uncertainty in treatment effectiveness, comparison with other policies should be made cautiously. We hope public health officials can use these findings to establish childcare exclusion policies that effectively interrupt disease transmission while minimizing economic consequences.

## Additional files

Additional file 1: **Appendix A.** "Methods and assumptions of the tool to evaluate the impact of exclusion policies associated with shigellosis". In this supplement, we provide further detail on the methods and assumptions used in the manuscript. (DOCX 81 kb)

Additional file 2: **Appendix B.** "A tool for the evaluation of shigellosis exclusion policies". In this supplement, the user may change the assumptions and see how that affects the results. (XLSX 122 kb)

Additional file 3: **Appendix C.** "Additional aggregate results for aggregated childcare days lost for various treatment mixes among the sample population potentially affected by a shigellosis outbreak". In this supplement, we provide additional results that can be calculated using the methods described in the paper, for a population of children. (DOCX 231 kb)

## Abbreviations

DNA: Deoxyribonucleic acid
PCR: Polymerase chain reaction
