# Supplementary material for: Evaluation of the impact of shigellosis exclusion policies in childcare settings upon detection of a shigellosis outbreak
Source: BMC Infect Dis. 2019 Feb 19;19:172. doi: 10.1186/s12879-019-3796-7 (PMC6379933; doi:10.1186/s12879-019-3796-7)
Supplement: Supplementary file 1 — Appendix A. "Methods and assumptions of the tool to evaluate the impact of exclusion policies associated with shigellosis". In this supplement, we provide further detail on the methods and assumptions used in the manuscript. (DOCX 81 kb) [file 12879_2019_3796_MOESM1_ESM.docx]

**Additional file 1: Appendix A. Methods and assumptions of the tool to evaluate the impact of exclusion policies associated with shigellosis.**

In this appendix, we provide additional details of the spreadsheet-based model to estimate the impact of exclusion policies associated with a shigellosis outbreak. Here we describe the default values we used for our estimations, based on the existing evidence. We also provide a spreadsheet for the user to adjust the model parameters based on specific local conditions.

Figure A1 provides another way to illustrate the different variables. Table A1 shows a summary of the calculations we used to estimate the probability that the patient remains infectious for the various exclusion policies we considered. Table A2 shows a summary of the calculations for the number of childcare-days lost per child.

**Figure A1.** Sequence of events for the four different types of treatment (panel a) and exclusion policies considered (panel b).

**a)**

**
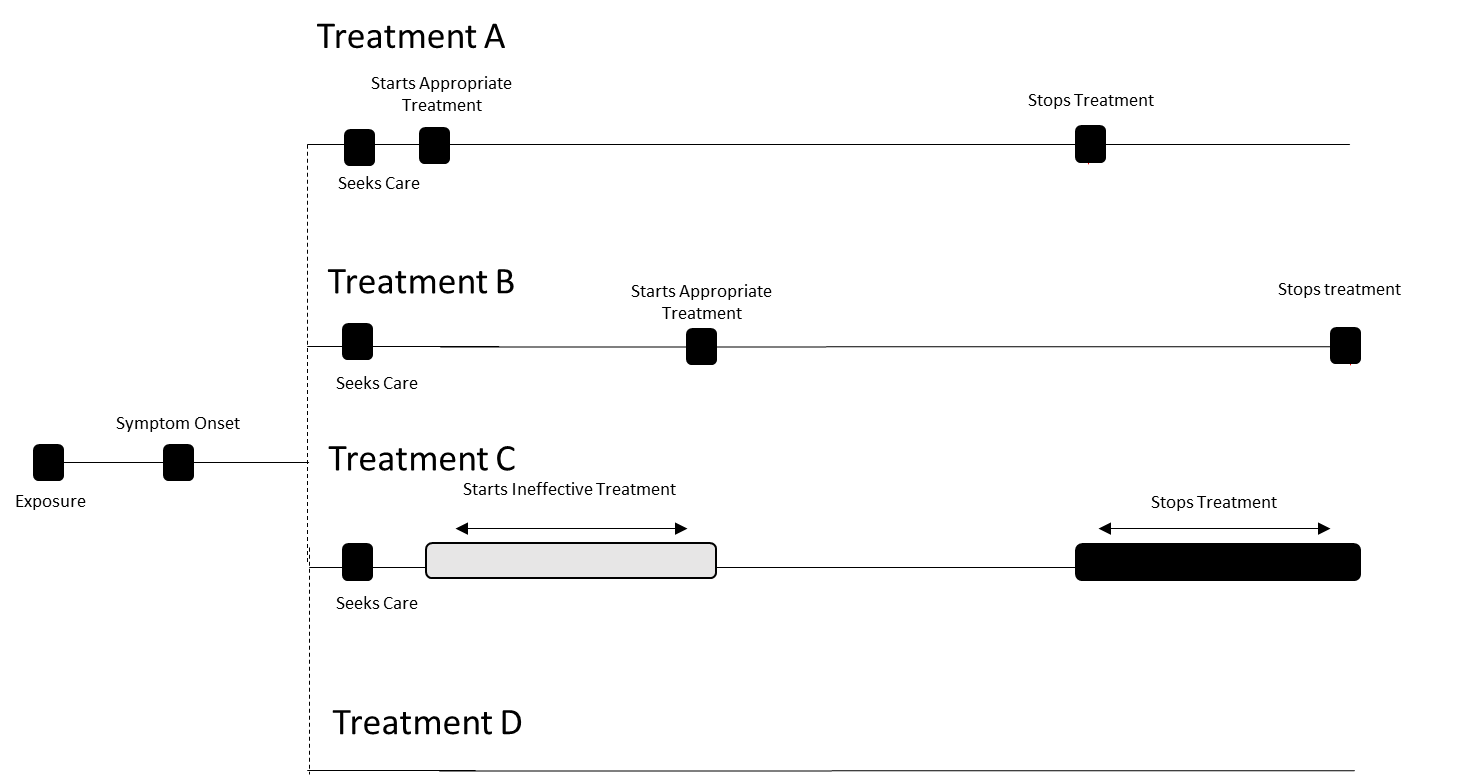
**

**b)**

**
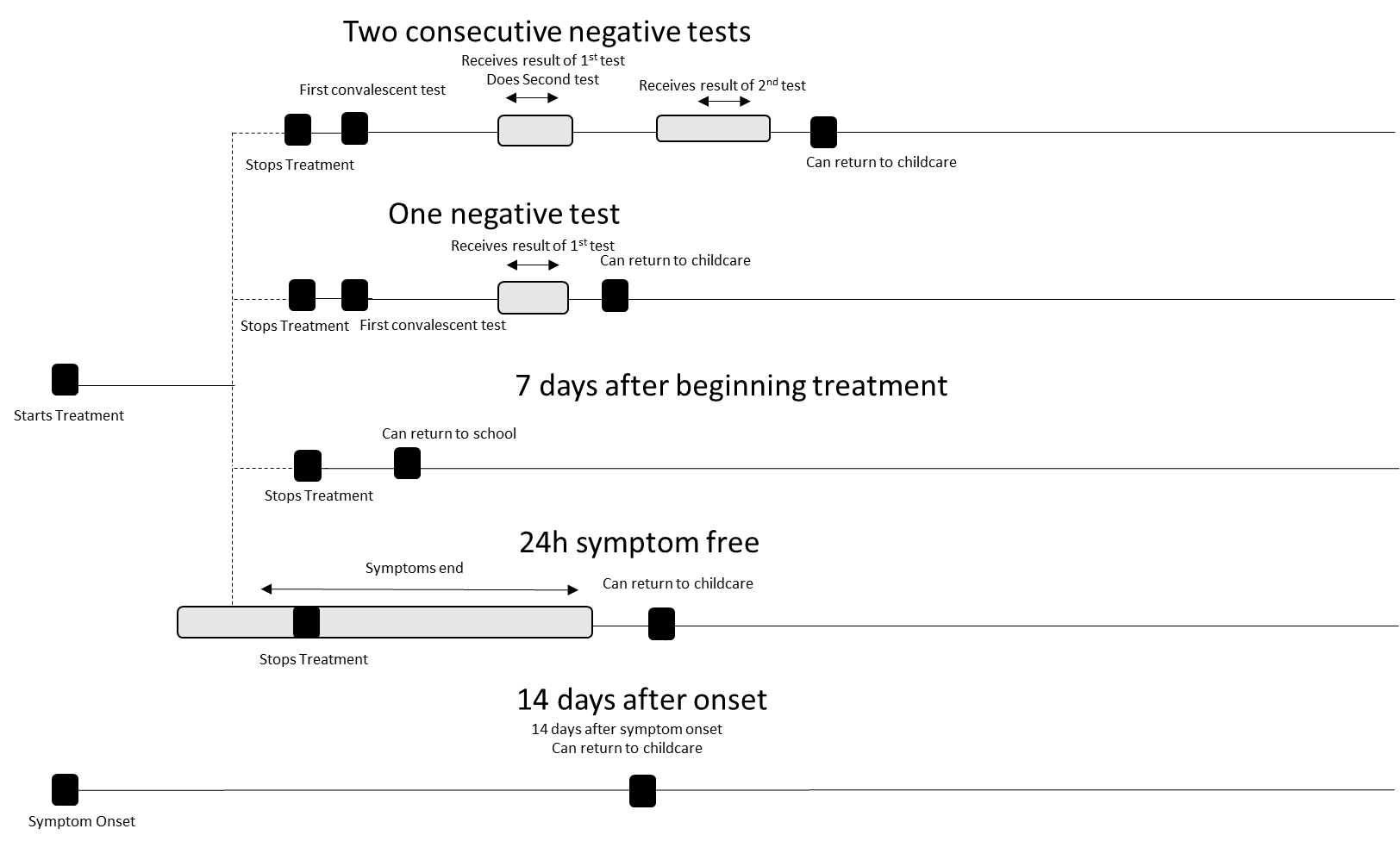
**

On panel a, we have not pinpointed the diagnosis time, since it can be made during a wide span of time, or, for treatment D, not at all. In any case, the determining constraints for duration of infectiousness are the start and effectiveness of treatment, and doctors may treat empirically in the absence of a secure diagnosis.

**Table A1.** Estimation methods of the probability that the excluded child remains infectious upon readmission, for different exclusion policies.

| Exclusion policy | Estimation method |
| --- | --- |
| 2 convalescent stool cultures yields negative results for *Shigella** | Probability of a false negative x probability of a false negative = (1-test’s sensitivity)^2^ |
| 2 PCR tests yield negative results* | Probability of a false negative x probability of a false negative= (1-test’s sensitivity)^2^ |
| 1 convalescent stool culture yields negative results for *Shigella* | Probability of a false negative = (1-test’s sensitivity) |
| 1 PCR tests yield negative results | Probability of a false negative = (1-test’s sensitivity) |
| 14 days after symptom onset, with no convalescent tests performed | Percent of patients remaining infectious 14 days after symptom onset |
| 7 days following beginning of antimicrobial treatment | If 7> maximum shedding duration, the probability the child remains infectious is 0;  If 7< minimum shedding duration, the probability the child remains infectious is 1;  If minimum shedding duration<7<maximum shedding duration, the probability the child remains infectious is (1-1/(maximum shedding duration – minimum shedding duration)*7) |
| 24 hours symptom-free and no convalescent tests | Share of patients remaining infectious 24 hours after symptoms stop |

* Our estimate also considered the duration of the testing process.

**Notes**: : For policies involving testing of convalescent stool cultures, the probability that the patient is infectious corresponds to Min(0,1-Day the child returns to school/Shedding duration)

**Table A2.** Estimation methods for the time a child with *Shigella* remains infectious upon readmission, for different exclusion policies. Exclusion policies were based on existing policies (1-6) and policies suggested by expert opinion.

| Exclusion policy | Estimation method |
| --- | --- |
| 2 convalescent stool cultures yield negative results | Treatments A, B, and C: Probability that the child returns to school infectious x (Duration of treatment + 2 x time to take the test and receive results) + Probability that the child returns to school recovered x Maximum (duration of treatment + 2 x time to take the test and receive results; shedding duration + 2 x time to take the test and receive results)  Treatment D: Probability that the child returns to school infectious x 2 x time to take the test and receive results + Probability that the child returns to school recovered x (shedding duration + 2 x time to take the test and receive results) |
| 2 PCR tests yield negative results | Treatments A, B, and C: Probability that the child returns to school infectious x (Duration of treatment + 2 x time to take the test and receive results) + Probability that the child returns to school recovered x Maximum (duration of treatment + 2 x time to take the test and receive results, shedding duration + 2 x time to take the test and receive results)  Treatment D: Probability that the child returns to school infectious x 2 x time to take the test and receive results + Probability that the child returns to school recovered x (shedding duration + 2 x time to take the test and receive results) |
| 1 laboratory analysis of convalescent stool samples yields negative results | Treatments A, B, and C: Probability that the child returns to school infectious x (Duration of treatment + time to take the test and receive results) + Probability that the child returns to school recovered x Maximum (duration of treatment + time to take the test and receive results, shedding duration + time to take the test and receive results)  Treatment D: Probability that the child returns to school infectious x time to take the test and receive results + Probability that the child returns to school recovered x (shedding duration + time to take the test and receive results) |
| 1 PCR tests yield negative results | Treatments A, B, and C: Probability that the child returns to school infectious x (Duration of treatment + time to take the test and receive results) + Probability that the child returns to school recovered x Maximum (duration of treatment + time to take the test and receive results, shedding duration + time to take the test and receive results)  Treatment D: Probability that the child returns to school infectious x time to take the test and receive results + Probability that the child returns to school recovered x (shedding duration + time to take the test and receive results) |
| 14 days after symptom onset, with no convalescent tests performed | 14 |
| 7 days following beginning of antimicrobial treatment | Duration of antimicrobial treatment + (7-duration of antimicrobial treatment) |
| 24 hours symptom-free and no convalescent tests | Symptom duration + 1 |

* Our estimate also considered the duration of the testing process.

**Notes**: For the policies involving testing of convalescent stool cultures, the probability that the child returns to school infectious corresponds to the probability of a false negative for policies involving one test, and to the squared probability of a false negative for policies involving two tests.

Because of the paucity of data for shigellosis outbreaks, we used the following assumptions in our model:

- There is a 1:1 correlation between positive results from the PCR and patients being infectious.
- A patient with shigellosis could fall into one of four different treatment scenarios, as defined in Table 1.
- We assumed default values of n=45 for setting size and an attack rate of 25%; however, these assumptions can be easily changed in the supplementary material.
- For exclusion policies that did not include "no treatment" patient type, we divided the distribution of "no treatment" patients equally among other types of patients.

**Bibliography for the Appendix**

1. Texas Department of State Health Services. Infectious Disease Control. Shigellosis. 2014 December 8 [cited 2015 August 26]; Available from: <https://www.dshs.state.tx.us/idcu/disease/shigellosis/>

2. State of Connecticut Department of Public Health. Shigellosis - Fact Sheet. 2015 April 9 [cited 2015 August 26]; Available from: <http://www.ct.gov/dph/cwp/view.asp?a=3136&q=388374>

3. Minnesota Department of Health. Specific Disease Exclusion Guidelines for Childcare and Preschool. 2015 July 29 [cited 2015 August 26]; Available from: <http://www.health.state.mn.us/divs/idepc/dtopics/foodborne/exclusions.html>

4. Colorado Department of Public Health & Environment. Shigellosis Outbreak Investigation & Control in Child Care Centers/Pre-schools. 2004 December [cited 2015 August 26]; Available from: <https://www.colorado.gov/pacific/sites/default/files/ComDis_CD-Shigellosis-Outbreak-Investigation-and-Control-in-Childcare-Centers_Preschools_0.pdf>

5. Turabelidze G, Bowen A, Lin M, Tucker A, Butler C, Fick F. Convalescent cultures for control of shigellosis outbreaks. The Pediatric infectious disease journal. 2010;29(8):728-30.

6. Shane AL, Tucker NA, Crump JA, Mintz ED, Painter JA. Sharing Shigella: risk factors for a multicommunity outbreak of shigellosis. Archives of Pediatrics & Adolescent Medicine. 2003;157(6):601-3.
