# Supplementary material for: Evaluation of the impact of shigellosis exclusion policies in childcare settings upon detection of a shigellosis outbreak
Source: BMC Infect Dis. 2019 Feb 19;19:172. doi: 10.1186/s12879-019-3796-7 (PMC6379933; doi:10.1186/s12879-019-3796-7)
Supplement: Supplementary file 3 — Appendix C. "Additional aggregate results for aggregated childcare days lost for various treatment mixes among the sample population potentially affected by a shigellosis outbreak". In this supplement, we provide additional results that can be calculated using the methods described in the paper, for a population of children. (DOCX 231 kb) [file 12879_2019_3796_MOESM3_ESM.docx]

**Additional file 3: Appendix C. Additional aggregate results for aggregated childcare days lost for various treatment mixes among the sample population potentially affected by a shigellosis outbreak.**

In this appendix, we explore the consequences of uncertainty regarding the treatment mixes in the patient population, and present results for different treatment mixes. The results were estimated for a public setting with a population of 45 individuals (including children and providers), consistent with a small childcare facility, and an attack rate of 25%. Instead of childcare-days lost per child, we present the aggregated childcare-days lost for a daycare population of 45 individuals, in which different individuals receive different treatments, per Table C1.

The different mixes are displayed in Table C1, and the results of the exclusion policies are displayed in Figs. C1 and C2.

**Table C1:** Sensitivity Analyses – Description of diverse treatment mixes among the patient population.

| Type of Treatment | Option 1 (%) | Option 2 (%) | Option 3 (%) |
| --- | --- | --- | --- |
| A. Immediate, effective treatment | 50% | 25% | 15% |
| B. Effective treatment after diagnosis | 40% | 25% | 15% |
| C. Ineffective treatment | 5% | 25% | 20% |
| D. No treatment | 5% | 25% | 50% |

**Notes:**

* For exclusion policies that do not include "no treatment" patient type, we divided the distribution of "no treatment" patients equally among other types of patients.

**Figure C1.** The impact of five different exclusion policies on aggregated childcare days missed and probability of infectiousness upon return to childcare (two exclusion policies were evaluated using Culture and PCR tests).


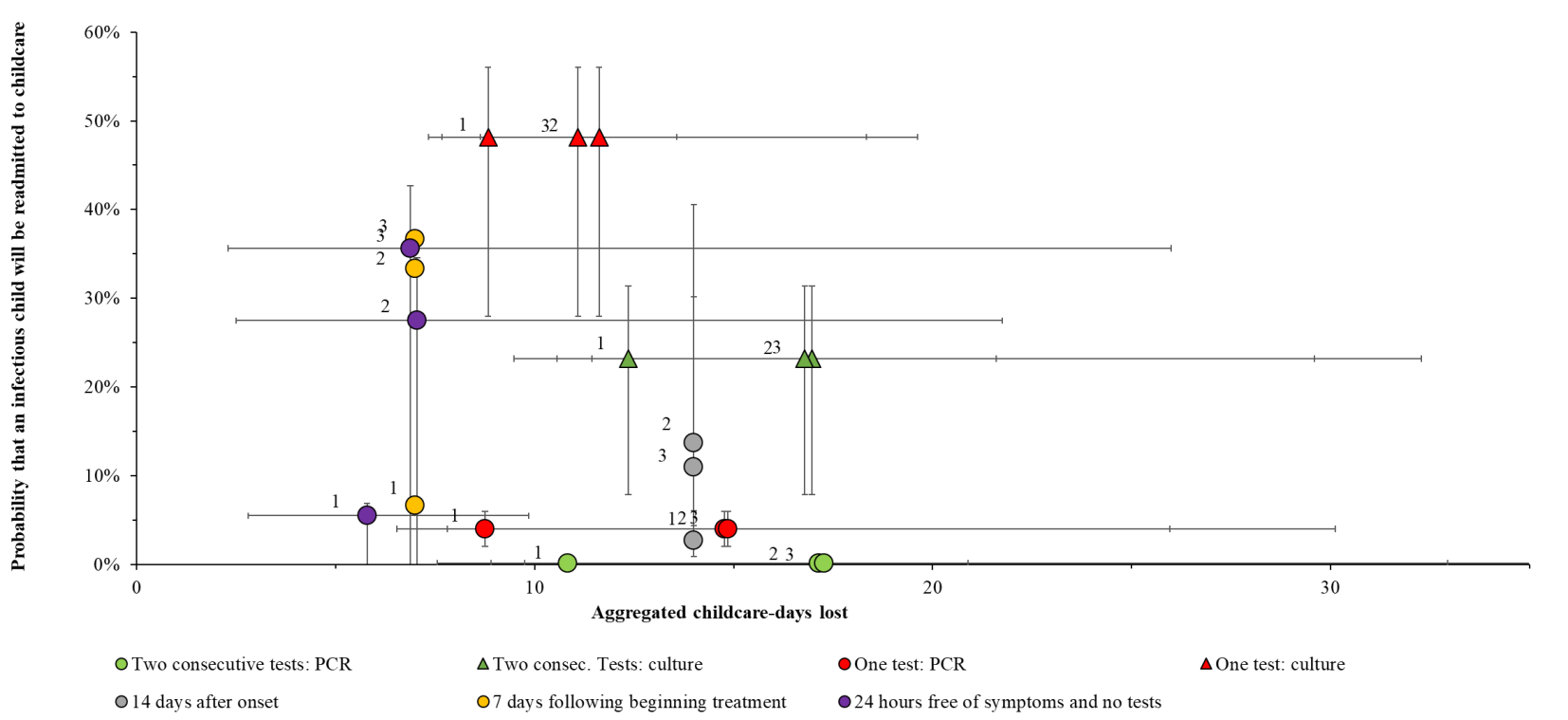


**Notes**: The points in the graph show the expected results for each of the five policies defined in Table 1 (i.e., two consecutive laboratory tests of stool yield negative results, one laboratory test of stools is negative, 14 days after symptom onset with no test performed, seven days after the beginning of antimicrobial treatment, and 24 hours free of symptoms with no test performed). Note that the figure shows two possible results for each of the two policies that require laboratory testing, based on whether the laboratory uses a PCR or culture-based diagnostic of *Shigella*.

The numbers 1, 2, 3 point to the specific combination of type of treatment that the patients in the childcare setting would have received, as shown in Table C1 (Option 1: 50% Immediate, effective treatment, 40% effective treatment after diagnosis, 5% ineffective treatment, and 5% no treatment; Option 2: 25% Immediate, effective treatment, 25% effective treatment after diagnosis, 25% ineffective treatment, and 25% no treatment; Option 3 15% Immediate, effective treatment, 15% effective treatment after diagnosis, 20% ineffective treatment, and 50% no treatment)

**Figure C2.** Total potential childcare-days lost in a shigellosis outbreak for each exclusion policy, for a setting of 45 children, and treatment mixes as in Table C1 (option 1 – panel a; option 2 – panel b; option 3 – panel c).


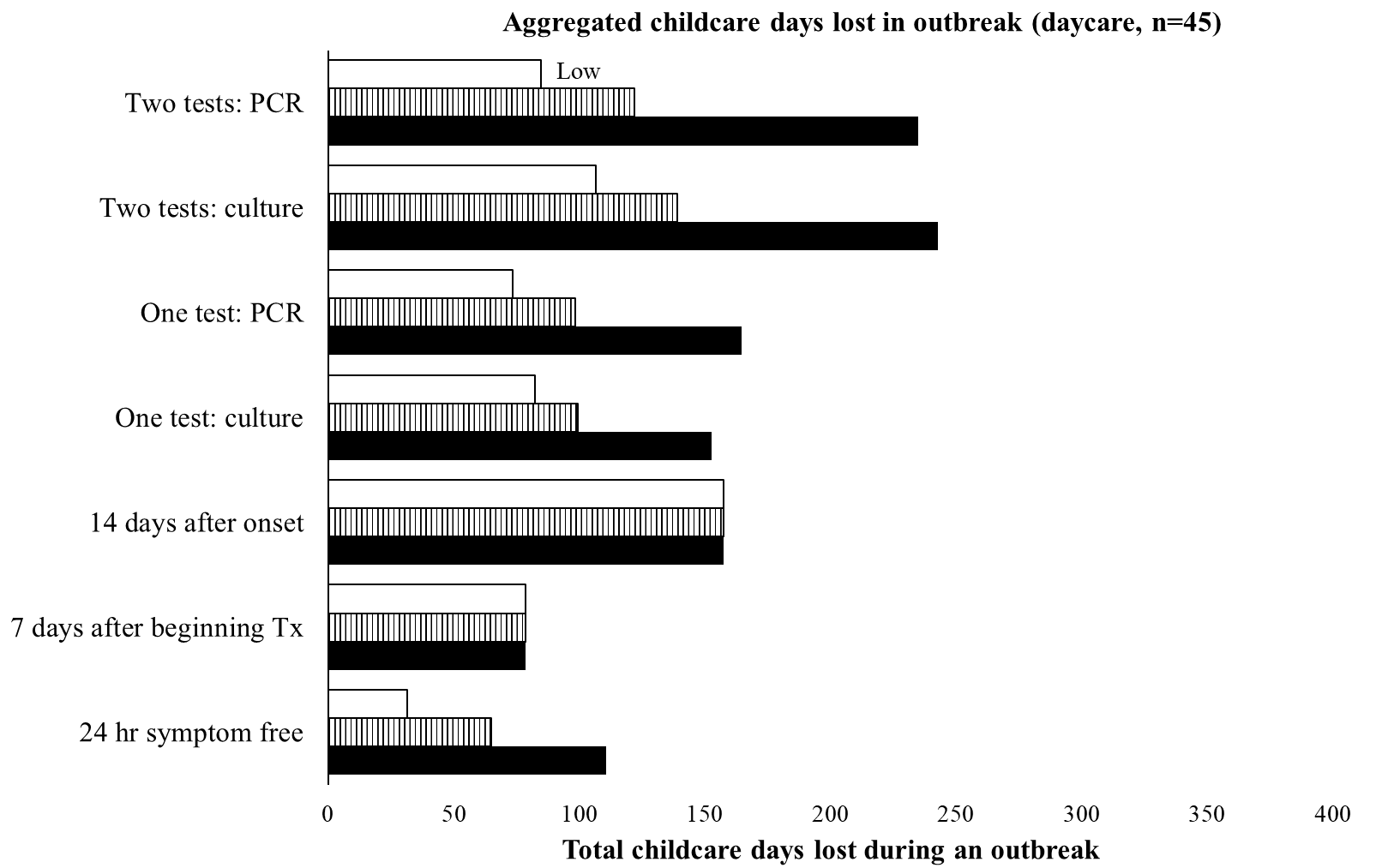


**a)**

**b)**

**
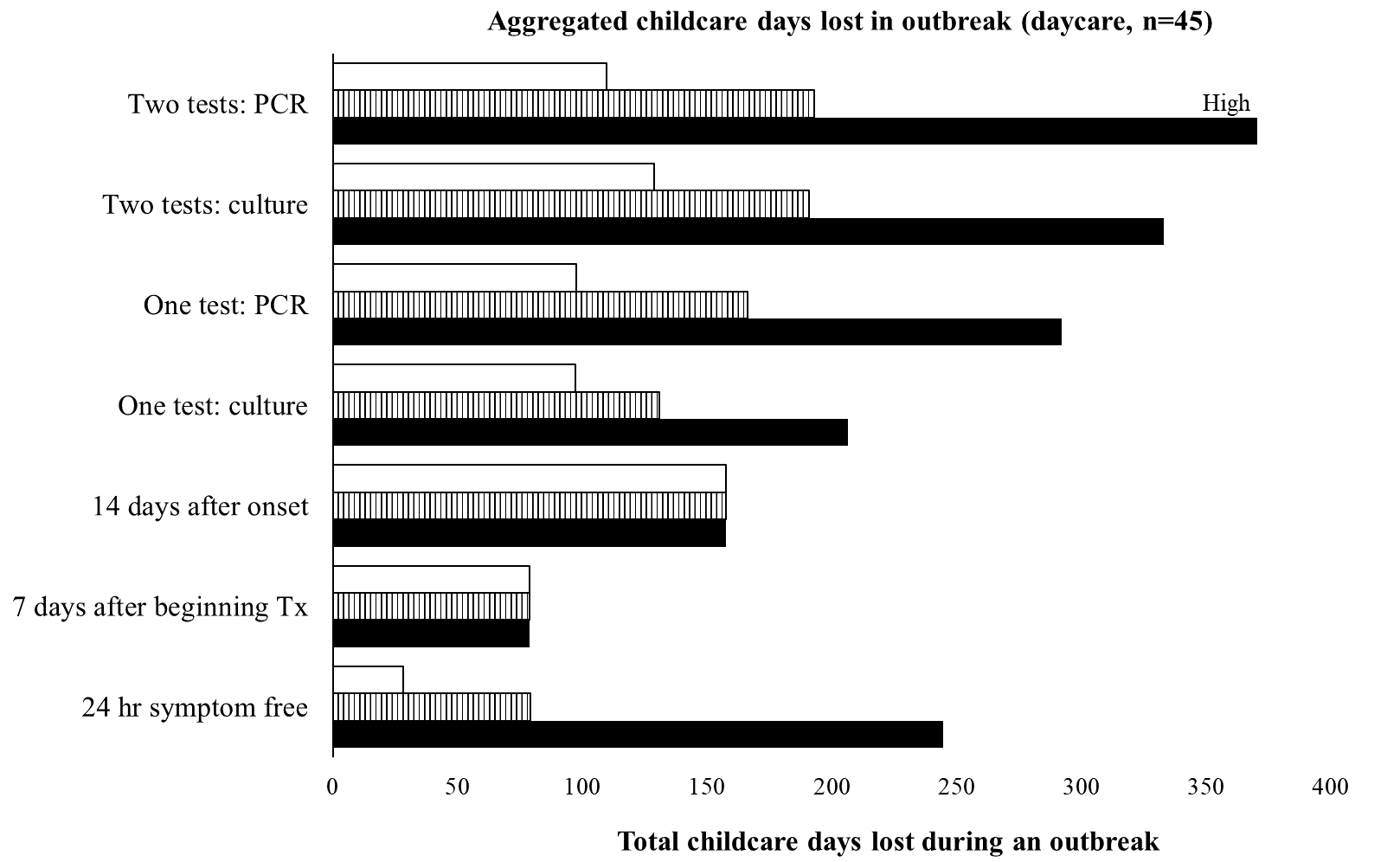
**

**c)**

**
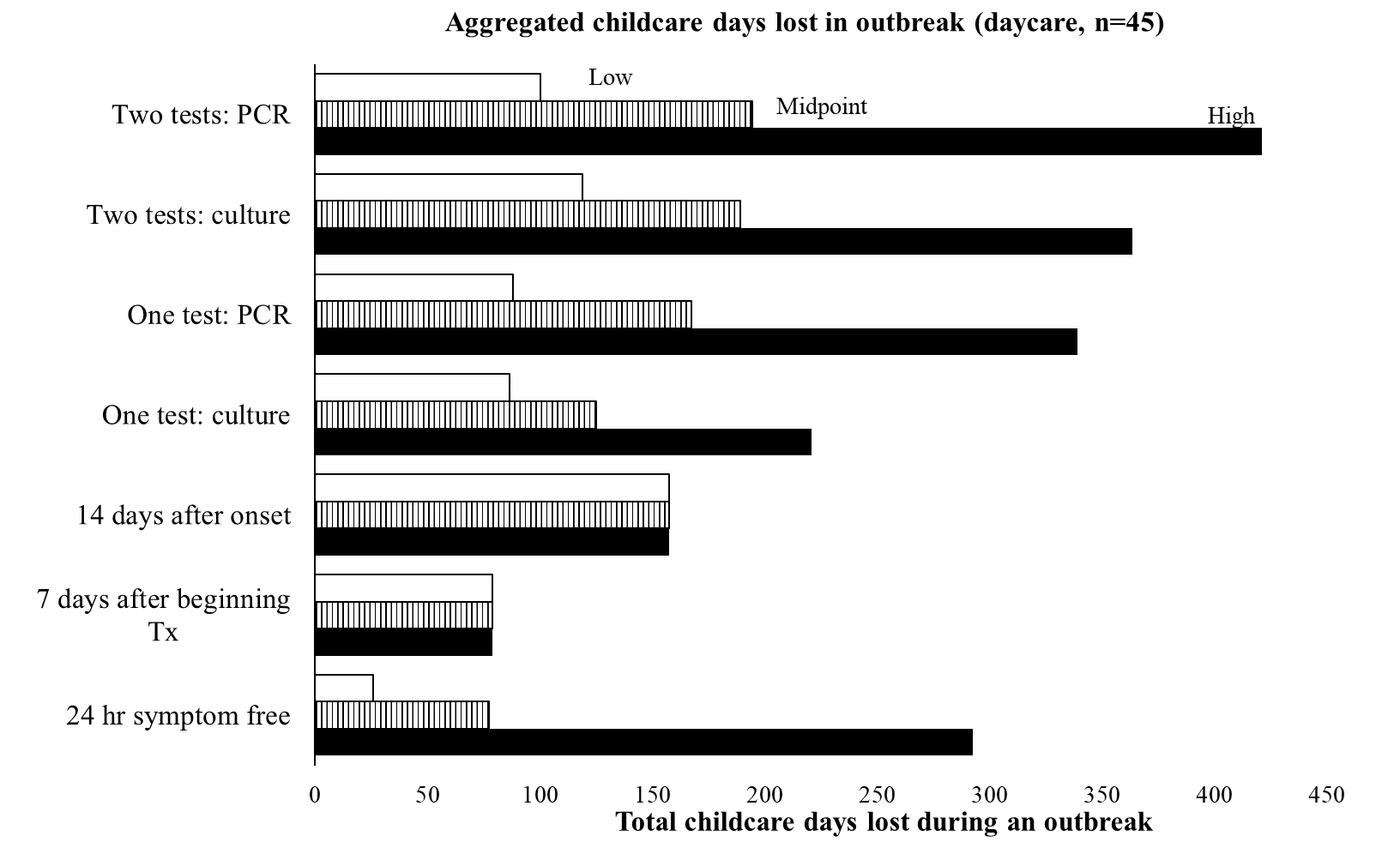
**

**Notes:** The bars in the graph show the expected results for each of the five policies defined in Table 1 (i.e., two consecutive laboratory tests of stool yield negative results, one laboratory test of stools is negative, 14 days after symptom onset with no test performed, seven days after the beginning of antimicrobial treatment, and 24 hours free of symptoms with no test performed). The three bars in each policy represent our most plausible estimation (midpoint), and a lower and upper range for total aggregate time lost in the childcare population. Note that the figure shows two possible results for each of the two policies that require laboratory testing, based on whether the laboratory uses a PCR or culture-based diagnostic of *Shigella*.

The letters a, b, and c refer to the specific combination of type of treatment that the patients in the childcare setting would have received, as shown in Table C1 (Panel A: 50% Immediate, effective treatment, 40% effective treatment after diagnosis, 5% ineffective treatment, and 5% no treatment; Panel B: 25% Immediate, effective treatment, 25% effective treatment after diagnosis, 25% ineffective treatment, and 25% no treatment; Panel C: 15% Immediate, effective treatment, 15% effective treatment after diagnosis, 20% ineffective treatment, and 50% no treatment).
